# (*R*)-(+)-3-Hydr­oxy-2-methoxy­carbonyl-8-methyl-8-azoniabicyclo­[3.2.1]octane l-bitartrate

**DOI:** 10.1107/S1600536808023532

**Published:** 2008-07-31

**Authors:** Jian-Bing Yu, Shuang-Wei Chen, Guo-Rong Zheng, Li-Yan Dai

**Affiliations:** a College of Materials Science and Chemical Engineering, Zhejiang University, Hangzhou 310027, People’s Republic of China; b Zhejiang Huayi Pharmaceutical Company, Yiwu, Zhejiang 322000, People’s Republic of China

## Abstract

(*RS*)-(±)-2-Methoxy­carbonyl-3-tropinone is an important inter­mediate for the preparation of cocaine and its derivatives. The molecule in the title compound, C_10_H_16_NO_3_^+^·C_4_H_5_O_6_^−^, is present as the enol tautomer. The six-membered ring adopts a half boat conformation, and the five-membered ring a slightly distorted envelope conformation. There are intra- and inter­molecular hydrogen bonds involving the hydroxyl, carboxyl groups and quaternary ammonium groups.

## Related literature

For related literature, see: Findlay (1957 ▶); Meltzer *et al.* (1994 ▶). 
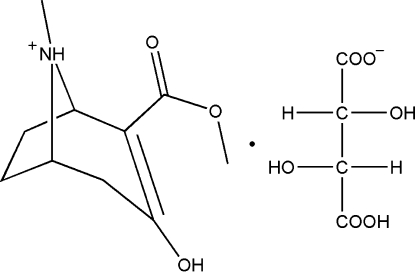


## Experimental

### 

#### Crystal data

C_10_H_16_NO_3_^+^·C_4_H_5_O_6_^−^
*M* *_r_* = 347.32
Monoclinic, 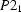

*a* = 6.5030 (10) Å
*b* = 15.914 (3) Å
*c* = 7.6626 (12) Å
β = 96.497 (3)°
*V* = 787.9 (2) Å^3^
*Z* = 2
Mo *K*α radiation
μ = 0.12 mm^−1^
*T* = 293 (2) K
0.50 × 0.49 × 0.37 mm

#### Data collection

SMART 1K CCD area-detector diffractometer
Absorption correction: multi-scan (**SADABS**; Sheldrick, 2002 ▶) *T*_min_ = 0.936, *T*_max_ = 0.961
4145 measured reflections
1522 independent reflections
1460 reflections with *I* > 2σ(*I*)
*R*_int_ = 0.082

#### Refinement

*R*[*F* ^2^ > 2σ(*F* ^2^)] = 0.064
*wR*(*F* ^2^) = 0.154
*S* = 1.05
1522 reflections
226 parameters
2 restraints
H atoms treated by a mixture of independent and constrained refinement
Δρ_max_ = 0.38 e Å^−3^
Δρ_min_ = −0.35 e Å^−3^

### 

Data collection: *SMART* (Bruker, 2001 ▶); cell refinement: *SAINT* (Bruker, 2001 ▶); data reduction: *SAINT*; program(s) used to solve structure: *SHELXTL* (Sheldrick, 2008 ▶); program(s) used to refine structure: *SHELXTL*; molecular graphics: *SHELXTL*; software used to prepare material for publication: *SHELXTL* and *publCIF* (Westrip, 2008 ▶).

## Supplementary Material

Crystal structure: contains datablocks global, I. DOI: 10.1107/S1600536808023532/rk2093sup1.cif

Structure factors: contains datablocks I. DOI: 10.1107/S1600536808023532/rk2093Isup2.hkl

Additional supplementary materials:  crystallographic information; 3D view; checkCIF report

## Figures and Tables

**Table 1:** Hydrogen-bond geometry (Å, °)

*D*—H⋯*A*	*D*—H	H⋯*A*	*D*⋯*A*	*D*—H⋯*A*
O1—H1*A*⋯O2	0.82	1.89	2.600 (4)	145
O7—H7⋯O9	0.82	2.06	2.562 (4)	120
N1—H1⋯O8^i^	0.954 (19)	1.82 (2)	2.724 (4)	158 (4)
O6—H6*A*⋯O2^ii^	0.82	2.58	3.182 (4)	132
O5—H5⋯O9^i^	0.82	1.72	2.535 (4)	172

Symmetry codes: (i) 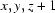; (ii) 
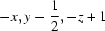
.

**Fig. 1.**
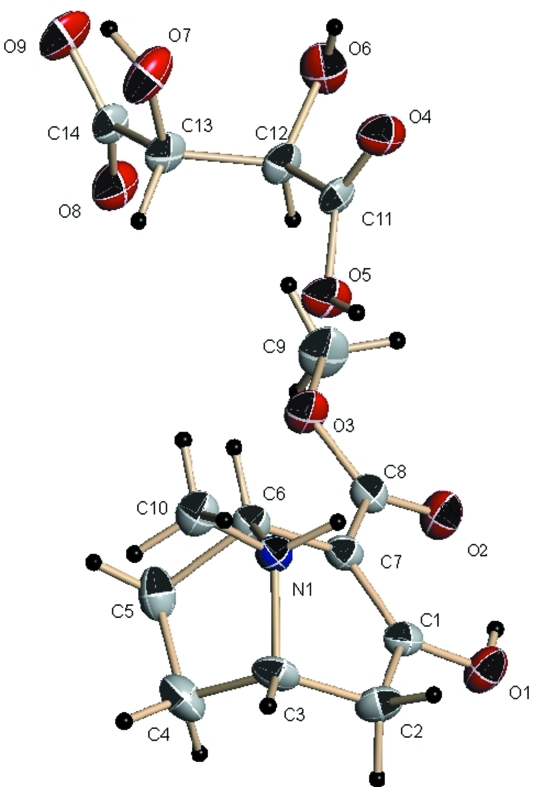
The molecular structure of I with the atom numbering scheme. Displacement ellipsoids are drawn at the 50% probability level. H atoms are presented as a small spheres of arbitrary radius.

**Fig. 2.**
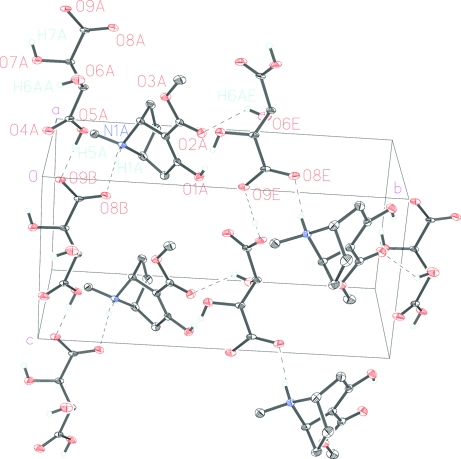
Parts of the two-dimensional network in I. H atoms not involved in hydrogen bonds have been omitted for clarity. Symmetry codes: (A) -x, y-1/2, -z+1; (B) x, y, z+1; (E) -x, y+1/2, -z+1.

## supplementary crystallographic information

### Comment

The (*RS*)-(±)-2-carbomethoxy-3-tropinone, I is an important intermediate for preparation of cocaine and its derivatives (Meltzer *et al.*, 1994). It could be resolved by recrystallizing its L- and *D*-bitartrates (Findlay, 1957). The molecular struture of I is the enol tautomer of the title compound. As shown on Fig. 1, the asymmetric unit of I contains a quaternary ammonium cation and a bitartrate. The 6-membered ring is nearly a chair conformation. The five-membered ring adopts nearly an envelope conformation with N1 atom deviation from C3/C4/C5/C6 plane 0.306 (2)Å. There are intra- and intermolecular hydrogen bonds involving the hydroxyl, carboxyl and quaternary ammonium ions. The system of these H-bond with formation a two-dimensional network presented on Fig. 2. All bond lengths and angles in I are normal.

### Experimental

All reagents were of analytical grade and used without further purification. The title compound I was prepared by the general procedure (Findlay, 1957). The single crystals were obtained by evaporation of its methanol solution.

### Refinement

H atoms were located in a difference Fourier map and refined isotropically with bond restraints N1–H1, other H atoms were positioned geometrically and treated as riding, with C–H and O–H bond lengths constrained to 0.96Å for methyl, 0.97Å for methylene, 0.98Å for methine and 0.82Å for hydroxyl, with *U*_iso_(H) = 1.5*U*_eq_(methyl C and hydroxyl O) and *U*_iso_(H) = 1.2*U*_eq_(methylene and methine C).

The 2813 Friedel pairs were merged.

### Crystal data

**Table:**

C_10_H_16_NO_3_^+^·C_4_H_5_O_6_^–^	*F*_000_ = 368
*M**_r_* = 347.32	*D*_x_ = 1.464 Mg m^−^^3^
Monoclinic, *P*2_1_	Mo *K*α radiation λ = 0.71073 Å
Hall symbol: P 2yb	Cell parameters from 2848 reflections
*a* = 6.5030 (10) Å	θ = 5.1–56.6º
*b* = 15.914 (3) Å	µ = 0.12 mm^−^^1^
*c* = 7.6626 (12) Å	*T* = 293 (2) K
β = 96.497 (3)º	Prism, colourless
*V* = 787.9 (2) Å^3^	0.50 × 0.49 × 0.37 mm
*Z* = 2	

### Data collection

**Table:**

SMART 1K CCD area-detector diffractometer	1522 independent reflections
Radiation source: Fine-focus sealed tube	1460 reflections with *I* > 2σ(*I*)
Monochromator: Graphite	*R*_int_ = 0.082
*T* = 293(2) K	θ_max_ = 25.5º
φ and ω scans	θ_min_ = 2.6º
Absorption correction: multi-scan(*SADABS*; Sheldrick, 2002)	*h* = −6→7
*T*_min_ = 0.936, *T*_max_ = 0.961	*k* = −19→19
4145 measured reflections	*l* = −9→8

### Refinement

**Table:**

Refinement on *F*^2^	Secondary atom site location: Difmap
Least-squares matrix: Full	Hydrogen site location: Geom
*R*[*F*^2^ > 2σ(*F*^2^)] = 0.064	H atoms treated by a mixture of independent and constrained refinement
*wR*(*F*^2^) = 0.154	*w* = 1/[σ^2^(*F*_o_^2^) + (0.1*P*)^2^] where *P* = (*F*_o_^2^ + 2*F*_c_^2^)/3
*S* = 1.05	(Δ/σ)_max_ < 0.001
1522 reflections	Δρ_max_ = 0.38 e Å^−^^3^
226 parameters	Δρ_min_ = −0.35 e Å^−^^3^
2 restraints	Extinction correction: None
Primary atom site location: Direct	

### Special details

**Table:**

Geometry. All s.u.'s (except the s.u. in the dihedral angle between two l.s. planes) are estimated using the full covariance matrix. The cell s.u.'s are taken into account individually in the estimation of s.u.'s in distances, angles and torsion angles; correlations between s.u.'s in cell parameters are only used when they are defined by crystal symmetry. An approximate (isotropic) treatment of cell s.u.'s is used for estimating s.u.'s involving l.s. planes.
Refinement. Refinement of *F*^2^ against ALL reflections. The weighted *R*-factor *wR* and goodness of fit *S* are based on *F*^2^, conventional *R*-factors *R* are based on *F*, with *F* set to zero for negative *F*^2^. The threshold expression of *F*^2^ > σ(*F*^2^) is used only for calculating *R*-factors(gt) *etc*. and is not relevant to the choice of reflections for refinement. *R*-factors based on *F*^2^ are statistically about twice as large as those based on *F*, and *R*-factors based on ALL data will be even larger.

### Fractional atomic coordinates and isotropic or equivalent isotropic displacement parameters (Å^2^)

**Table:**

	*x*	*y*	*z*	*U*_iso_*/*U*_eq_	
O1	0.4137 (5)	0.42707 (19)	1.0979 (4)	0.0430 (7)	
H1A	0.3313	0.4411	1.0140	0.064*	
O2	0.2485 (5)	0.42415 (18)	0.7727 (4)	0.0424 (7)	
O3	0.3452 (4)	0.31950 (18)	0.6043 (3)	0.0394 (6)	
O4	0.0156 (5)	0.0122 (2)	0.7351 (4)	0.0490 (7)	
O5	0.2813 (4)	0.0965 (2)	0.8123 (3)	0.0443 (7)	
H5	0.2735	0.0767	0.9101	0.066*	
O6	−0.0490 (4)	0.0881 (2)	0.4144 (4)	0.0503 (7)	
H6A	−0.0971	0.0419	0.4346	0.076*	
O7	0.2769 (6)	−0.03238 (19)	0.4196 (4)	0.0533 (8)	
H7	0.2713	−0.0479	0.3171	0.080*	
O8	0.3679 (5)	0.16640 (19)	0.2317 (4)	0.0470 (7)	
O9	0.2908 (5)	0.0395 (2)	0.1220 (3)	0.0494 (8)	
N1	0.6693 (4)	0.20164 (19)	1.0231 (4)	0.0285 (6)	
C1	0.5207 (5)	0.3624 (2)	1.0542 (5)	0.0302 (7)	
C2	0.6708 (6)	0.3287 (2)	1.1994 (5)	0.0387 (9)	
H2A	0.5951	0.3054	1.2902	0.046*	
H2B	0.7562	0.3743	1.2509	0.046*	
C3	0.8082 (5)	0.2615 (3)	1.1353 (5)	0.0356 (8)	
H3	0.8852	0.2319	1.2341	0.043*	
C4	0.9545 (5)	0.2942 (3)	1.0073 (6)	0.0483 (10)	
H4A	1.0871	0.2658	1.0263	0.058*	
H4B	0.9771	0.3541	1.0230	0.058*	
C5	0.8474 (6)	0.2756 (3)	0.8221 (6)	0.0437 (9)	
H5A	0.8467	0.3249	0.7475	0.052*	
H5B	0.9156	0.2299	0.7678	0.052*	
C6	0.6273 (5)	0.2510 (2)	0.8544 (5)	0.0304 (7)	
H6	0.5596	0.2165	0.7587	0.036*	
C7	0.4975 (5)	0.3252 (2)	0.8921 (5)	0.0298 (7)	
C8	0.3528 (5)	0.3622 (2)	0.7538 (5)	0.0304 (7)	
C9	0.2115 (7)	0.3550 (3)	0.4589 (6)	0.0508 (11)	
H9A	0.0828	0.3717	0.4985	0.076*	
H9B	0.1856	0.3137	0.3677	0.076*	
H9C	0.2774	0.4031	0.4139	0.076*	
C10	0.7663 (6)	0.1188 (2)	0.9999 (5)	0.0390 (8)	
H10A	0.6943	0.0908	0.9003	0.058*	
H10B	0.7591	0.0854	1.1033	0.058*	
H10C	0.9086	0.1267	0.9812	0.058*	
C11	0.1363 (5)	0.0638 (2)	0.7001 (4)	0.0305 (7)	
C12	0.1436 (5)	0.0981 (2)	0.5152 (4)	0.0334 (8)	
H12	0.1752	0.1582	0.5240	0.040*	
C13	0.3152 (5)	0.0546 (2)	0.4291 (4)	0.0327 (8)	
H13	0.4475	0.0643	0.5010	0.039*	
C14	0.3271 (5)	0.0916 (3)	0.2461 (4)	0.0321 (7)	
H1	0.543 (4)	0.188 (2)	1.068 (5)	0.026 (9)*	

### Atomic displacement parameters (Å^2^)

**Table:**

	*U*^11^	*U*^22^	*U*^33^	*U*^12^	*U*^13^	*U*^23^
O1	0.0541 (15)	0.0441 (14)	0.0301 (14)	0.0102 (13)	0.0021 (11)	−0.0035 (13)
O2	0.0453 (13)	0.0446 (14)	0.0360 (15)	0.0180 (12)	−0.0015 (11)	0.0030 (13)
O3	0.0432 (13)	0.0527 (15)	0.0200 (13)	0.0097 (12)	−0.0066 (9)	0.0019 (12)
O4	0.0579 (15)	0.0664 (18)	0.0237 (13)	−0.0218 (15)	0.0089 (11)	−0.0036 (14)
O5	0.0545 (14)	0.0633 (16)	0.0141 (12)	−0.0159 (14)	0.0000 (10)	0.0055 (13)
O6	0.0430 (13)	0.079 (2)	0.0269 (14)	0.0105 (15)	−0.0062 (10)	0.0089 (15)
O7	0.089 (2)	0.0441 (15)	0.0291 (16)	0.0119 (16)	0.0166 (15)	0.0036 (13)
O8	0.0640 (17)	0.0522 (17)	0.0267 (15)	−0.0055 (14)	0.0139 (12)	0.0041 (13)
O9	0.0798 (19)	0.0564 (16)	0.0124 (12)	0.0012 (16)	0.0067 (12)	0.0005 (12)
N1	0.0293 (12)	0.0377 (15)	0.0176 (13)	−0.0008 (11)	−0.0007 (10)	0.0038 (12)
C1	0.0320 (15)	0.0308 (16)	0.0272 (19)	−0.0059 (14)	0.0012 (12)	0.0005 (14)
C2	0.0456 (18)	0.044 (2)	0.0238 (18)	−0.0020 (17)	−0.0083 (14)	−0.0023 (17)
C3	0.0320 (15)	0.0437 (18)	0.0277 (18)	−0.0068 (15)	−0.0114 (13)	0.0067 (16)
C4	0.0299 (17)	0.057 (2)	0.058 (3)	−0.0043 (18)	0.0027 (15)	0.007 (2)
C5	0.0405 (18)	0.048 (2)	0.045 (2)	0.0075 (16)	0.0155 (15)	0.0118 (19)
C6	0.0323 (15)	0.0375 (18)	0.0207 (17)	−0.0010 (14)	0.0000 (12)	0.0056 (14)
C7	0.0285 (14)	0.0363 (16)	0.0235 (17)	−0.0033 (13)	−0.0010 (11)	0.0032 (14)
C8	0.0294 (14)	0.0359 (17)	0.0255 (19)	−0.0012 (14)	0.0018 (13)	0.0058 (14)
C9	0.055 (2)	0.072 (3)	0.0219 (19)	0.016 (2)	−0.0107 (16)	0.007 (2)
C10	0.0442 (18)	0.0422 (19)	0.0292 (19)	0.0088 (16)	−0.0010 (14)	0.0049 (16)
C11	0.0374 (15)	0.0406 (17)	0.0138 (16)	−0.0002 (14)	0.0043 (11)	−0.0035 (15)
C12	0.0439 (17)	0.0420 (18)	0.0140 (16)	0.0032 (16)	0.0026 (13)	0.0020 (15)
C13	0.0422 (17)	0.0451 (19)	0.0103 (16)	0.0031 (15)	0.0000 (12)	0.0035 (14)
C14	0.0382 (16)	0.046 (2)	0.0118 (15)	0.0002 (15)	0.0041 (11)	0.0018 (15)

### Geometric parameters (Å, °)

**Table:**

O1—C1	1.307 (5)	C3—C4	1.533 (6)
O1—H1A	0.8200	C3—H3	0.9800
O2—C8	1.215 (4)	C4—C5	1.537 (7)
O3—C8	1.328 (5)	C4—H4A	0.9700
O3—C9	1.448 (4)	C4—H4B	0.9700
O4—C11	1.188 (5)	C5—C6	1.531 (5)
O5—C11	1.309 (4)	C5—H5A	0.9700
O5—H5	0.8200	C5—H5B	0.9700
O6—C12	1.404 (4)	C6—C7	1.498 (5)
O6—H6A	0.8200	C6—H6	0.9800
O7—C13	1.407 (5)	C7—C8	1.459 (5)
O7—H7	0.8200	C9—H9A	0.9600
O8—C14	1.228 (5)	C9—H9B	0.9600
O9—C14	1.262 (5)	C9—H9C	0.9600
N1—C10	1.481 (5)	C10—H10A	0.9600
N1—C6	1.511 (4)	C10—H10B	0.9600
N1—C3	1.511 (4)	C10—H10C	0.9600
N1—H1	0.954 (19)	C11—C12	1.524 (4)
C1—C7	1.369 (5)	C12—C13	1.525 (5)
C1—C2	1.495 (5)	C12—H12	0.9800
C2—C3	1.511 (6)	C13—C14	1.531 (4)
C2—H2A	0.9700	C13—H13	0.9800
C2—H2B	0.9700		
			
C1—O1—H1A	109.5	N1—C6—C5	100.9 (3)
C8—O3—C9	115.2 (3)	C7—C6—H6	111.8
C11—O5—H5	109.5	N1—C6—H6	111.8
C12—O6—H6A	109.5	C5—C6—H6	111.8
C13—O7—H7	109.5	C1—C7—C8	118.7 (3)
C10—N1—C6	113.5 (3)	C1—C7—C6	120.6 (3)
C10—N1—C3	113.2 (3)	C8—C7—C6	120.6 (3)
C6—N1—C3	101.4 (3)	O2—C8—O3	123.4 (3)
C10—N1—H1	103 (2)	O2—C8—C7	124.4 (3)
C6—N1—H1	111 (2)	O3—C8—C7	112.2 (3)
C3—N1—H1	115 (2)	O3—C9—H9A	109.5
O1—C1—C7	124.5 (3)	O3—C9—H9B	109.5
O1—C1—C2	114.5 (3)	H9A—C9—H9B	109.5
C7—C1—C2	121.0 (3)	O3—C9—H9C	109.5
C1—C2—C3	111.9 (3)	H9A—C9—H9C	109.5
C1—C2—H2A	109.2	H9B—C9—H9C	109.5
C3—C2—H2A	109.2	N1—C10—H10A	109.5
C1—C2—H2B	109.2	N1—C10—H10B	109.5
C3—C2—H2B	109.2	H10A—C10—H10B	109.5
H2A—C2—H2B	107.9	N1—C10—H10C	109.5
C2—C3—N1	107.1 (3)	H10A—C10—H10C	109.5
C2—C3—C4	113.6 (3)	H10B—C10—H10C	109.5
N1—C3—C4	103.0 (3)	O4—C11—O5	124.9 (3)
C2—C3—H3	111.0	O4—C11—C12	123.2 (3)
N1—C3—H3	111.0	O5—C11—C12	111.8 (3)
C4—C3—H3	111.0	O6—C12—C11	110.6 (3)
C3—C4—C5	106.0 (3)	O6—C12—C13	111.2 (3)
C3—C4—H4A	110.5	C11—C12—C13	110.0 (3)
C5—C4—H4A	110.5	O6—C12—H12	108.3
C3—C4—H4B	110.5	C11—C12—H12	108.3
C5—C4—H4B	110.5	C13—C12—H12	108.3
H4A—C4—H4B	108.7	O7—C13—C12	109.5 (3)
C6—C5—C4	103.5 (3)	O7—C13—C14	111.0 (3)
C6—C5—H5A	111.1	C12—C13—C14	109.7 (3)
C4—C5—H5A	111.1	O7—C13—H13	108.9
C6—C5—H5B	111.1	C12—C13—H13	108.9
C4—C5—H5B	111.1	C14—C13—H13	108.9
H5A—C5—H5B	109.0	O8—C14—O9	126.3 (3)
C7—C6—N1	107.2 (3)	O8—C14—C13	119.2 (3)
C7—C6—C5	112.8 (3)	O9—C14—C13	114.4 (3)
			
O1—C1—C2—C3	−172.8 (3)	C5—C6—C7—C1	−77.6 (4)
C7—C1—C2—C3	9.1 (5)	N1—C6—C7—C8	−151.1 (3)
C1—C2—C3—N1	−47.1 (4)	C5—C6—C7—C8	98.7 (4)
C1—C2—C3—C4	65.9 (4)	C9—O3—C8—O2	3.7 (5)
C10—N1—C3—C2	−160.7 (3)	C9—O3—C8—C7	−177.3 (3)
C6—N1—C3—C2	77.4 (3)	C1—C7—C8—O2	−0.7 (5)
C10—N1—C3—C4	79.3 (3)	C6—C7—C8—O2	−177.0 (3)
C6—N1—C3—C4	−42.6 (3)	C1—C7—C8—O3	−179.7 (3)
C2—C3—C4—C5	−96.8 (4)	C6—C7—C8—O3	3.9 (4)
N1—C3—C4—C5	18.7 (4)	O4—C11—C12—O6	23.5 (5)
C3—C4—C5—C6	11.9 (4)	O5—C11—C12—O6	−158.1 (3)
C10—N1—C6—C7	170.3 (3)	O4—C11—C12—C13	−99.7 (4)
C3—N1—C6—C7	−68.0 (3)	O5—C11—C12—C13	78.7 (4)
C10—N1—C6—C5	−71.4 (4)	O6—C12—C13—O7	−62.8 (4)
C3—N1—C6—C5	50.2 (3)	C11—C12—C13—O7	60.0 (4)
C4—C5—C6—C7	76.1 (4)	O6—C12—C13—C14	59.3 (4)
C4—C5—C6—N1	−37.9 (4)	C11—C12—C13—C14	−177.9 (3)
O1—C1—C7—C8	3.8 (5)	O7—C13—C14—O8	−177.1 (3)
C2—C1—C7—C8	−178.4 (3)	C12—C13—C14—O8	61.7 (4)
O1—C1—C7—C6	−179.8 (3)	O7—C13—C14—O9	3.4 (4)
C2—C1—C7—C6	−2.0 (5)	C12—C13—C14—O9	−117.8 (3)
N1—C6—C7—C1	32.6 (4)		

### Hydrogen-bond geometry (Å, °)

**Table:**

*D*—H···*A*	*D*—H	H···*A*	*D*···*A*	*D*—H···*A*
O1—H1A···O2	0.82	1.89	2.600 (4)	145
O7—H7···O9	0.82	2.06	2.562 (4)	120
N1—H1···O8^i^	0.954 (19)	1.82 (2)	2.724 (4)	158 (4)
O6—H6A···O2^ii^	0.82	2.58	3.182 (4)	132
O5—H5···O9^i^	0.82	1.72	2.535 (4)	172

Symmetry codes: (i) *x*, *y*, *z*+1; (ii) −*x*, *y*−1/2, −*z*+1.
